# A Bayesian network structure learning approach to identify genes associated with stress in spleens of chickens

**DOI:** 10.1038/s41598-022-11633-7

**Published:** 2022-05-06

**Authors:** E. A. Videla Rodriguez, John B. O. Mitchell, V. Anne Smith

**Affiliations:** 1 School of Biology, University of St Andrews, St Andrews, KY16 9TH Fife UK; 2 EaStCHEM School of Chemistry and Biomedical Sciences Research Complex, University of St Andrews, St Andrews, KY16 9ST Fife UK

**Keywords:** Computational biology and bioinformatics, Data mining, Animal breeding, Gene expression

## Abstract

Differences in the expression patterns of genes have been used to measure the effects of non-stress or stress conditions in poultry species. However, the list of genes identified can be extensive and they might be related to several biological systems. Therefore, the aim of this study was to identify a small set of genes closely associated with stress in a poultry animal model, the chicken (*Gallus gallus*), by reusing and combining data previously published together with bioinformatic analysis and Bayesian networks in a multi-step approach. Two datasets were collected from publicly available repositories and pre-processed. Bioinformatics analyses were performed to identify genes common to both datasets that showed differential expression patterns between non-stress and stress conditions. Bayesian networks were learnt using a Simulated Annealing algorithm implemented in the software Banjo. The structure of the Bayesian network consisted of 16 out of 19 genes together with the stress condition. Network structure showed CARD19 directly connected to the stress condition plus highlighted CYGB, BRAT1, and EPN3 as relevant, suggesting these genes could play a role in stress. The biological functionality of these genes is related to damage, apoptosis, and oxygen provision, and they could potentially be further explored as biomarkers of stress.

## Introduction

Stress has been defined as the physiological response triggered by an external or internal stimulus, with the aim of coping with or dealing with the stimulus^1,2^. The stress response is perceived, integrated, and displayed in the interplay of three major systems, the nervous system, the endocrine system, and the immune system, also known as the immune-neuroendocrine (INE) interplay^3–5^. Two axes of the INE interplay play the major roles in the stress response during the initial exposure to a stimulus: the Adrenergic Nervous System and the Hypothalamus-Pituitary-Adrenergic Axis are in charge of the fight or flight response and the activation of metabolic processes such as gluconeogenesis or proteolysis, respectively^6–9^. The activation of these two axes translates into a series of general and specific physiological as well as behavioral adaptations to deal with the influence of the potentially stressful stimulus^3,10,11^.

The stress response consists of a general as well as a specific response to a potentially stressful stimulus. While the general response is independent of the nature of the stimulus, the specific response is mostly dependent on the type of stimulus^3,10,12,13^. For example, a bird facing a potential predator might either display an escape behaviour, running as fast as possible from the predator, or display freezing behaviour, pretending to be dead by showing a tonic immobility response to avoid the threat. As another example, a bird under heat stress conditions can display behaviours such as panting or wing spreading to dissipate as much excess heat as possible^12,14^. In terms of the physiology, the exposure of birds to chronic heat stress can affect many systems, such as the digestive tract, the gut microbiota, the immune system, and the antioxidant system among others^15–18^. In the complexity of the stress response, and in particular heat stress, some of the effects of the stress response can have indirect consequences on other systems. To some extent, poor productive performance in poultry (e.g., poor body weight gain or egg production) can be associated with a lower feed intake as a consequence of the exposure to heat stress, or the alteration of the structure of immune organs can be associated with the imbalance of the oxidant/anti-oxidant status as a result of oxidative stress^3,18–20^.

Considering the complexity of the stress phenomenon, many indicators have been used to study the stress response. Corticosterone is considered as the main indicator of stress, as increased levels have been shown by birds exposed to stress protocols^21–23^. However, other indicators have been studied, such as changes in the microbial communities of the gut, immune suppression, oxidative status, and the morphology of the digestive tract among others^17,20,21,24,25^. Changes in the expression patterns of genes driven by the exposure to a potentially stressful stimulus have also being studied^26,27^. This indicator combines high-throughput technologies to measure the gene expression with bioinformatic tools to identify statistically significant differences between birds raised under non-stress conditions and birds exposed to the stressful stimulus^28–31^. Even though the set of significant genes can be used to identify molecular pathways associated with stress, one of the main drawbacks of this approach is that the number of statistically significant genes can be quite extensive and they can be related to several mechanisms and systems within the physiology of the birds^29,32,33^.

We can solve this problem with further exploration to discover hidden patterns behind the data: key genes among these lists that are most relevant to the stress condition of interest can be identified using Bayesian networks^34–36^. Bayesian networks represent a powerful mathematical tool that provide new insights by identifying probabilistic relationships between a given set of variables, distinguishing direct from indirect influences^35,37–39^. A Bayesian network is a directed acyclic graph (DAG), consisting of a set of nodes, that represent each of the variables, and edges, that display the relationships between the nodes^40,41^. Because the network is acyclic, i.e., has no loops, relationships among variables within a Bayesian network are often described using a ‘family analogy’ where a node of interest can have parents and children, considering those nodes with edges coming into or going out of the node of interest, respectively. Additionally, spouses are those nodes that share a common child with the node of interest^40,42^. The set of parents, children, and spouses represents one of the main properties of Bayesian networks known as a Markov Blanket. Considering that Bayesian networks are based on probability theory, this property allows identification of a small set of nodes in close association with a node of interest that makes this node probabilistically independent from the rest of the network^40,42^.

In this complex scenario, the aim of this study was to identify a small set of genes in close association with the stress condition in a poultry animal model, the chicken (*Gallus gallus*). We further increased the statistical power of our method by reusing and combining data from separate experiments combined with the accuracy of bioinformatic analysis and the power of Bayesian networks in a multi-step interdisciplinary approach. Publicly available repositories were explored to collect data coming from two experiments where the expression values of genes were measured in the spleen of chickens exposed to heat stress by RNA-sequencing. Bioinformatic analyses were implemented to identify a set of genes driven by the exposure to the stressor, followed by learning the structure of a Bayesian network to display the relationships and interactions between the genes and the stressful condition. The structure of the network was divided into communities of densely connected nodes, with a special focus on the community of nodes related to the stress condition. Finally, the biological meaning of the discovered interactions and relationships was explored.

## Results

**Figure 1:**
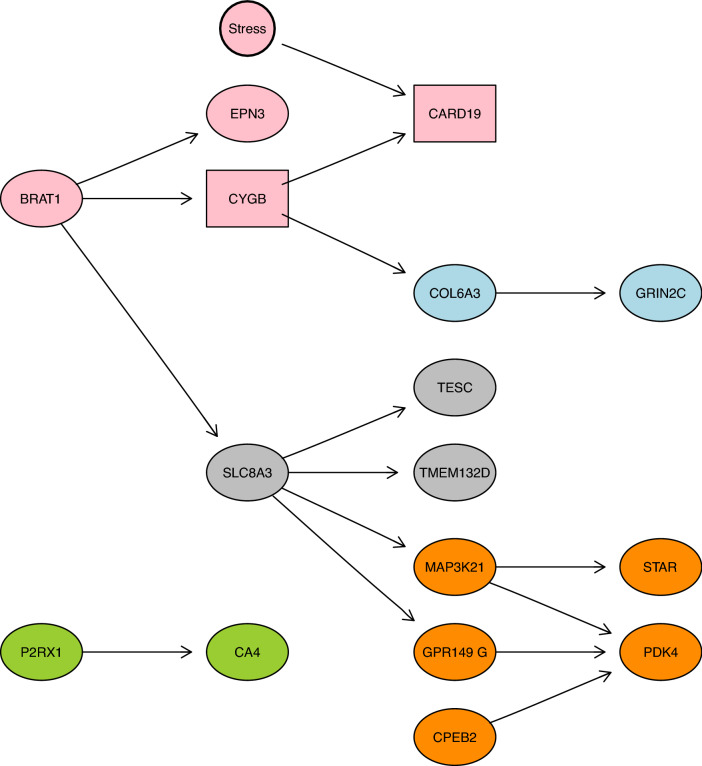
Bayesian network and community analysis of a set of genes. 19 genes showing differences in expression pattern were initially included at the time of learning the structure of the network in addition to the stressful condition; however, only 16 out of those 19 were linked in a network structure. Nodes represent each one of the genes and the stressful condition (circle-shaped node, thick outline), the edges represent probabilistic dependencies between the nodes. Note the direction of the arrows do not represent causation, but instead a statistical relationship. The Markov Blanket of the stress condition (rectangle-shaped nodes) consisted of two genes, CARD19 (child) and CYGB (spouse). Five communities of densely connected nodes were identified (different colours represent different communities). The community of the condition consisted of 4 genes (CARD19, EPN3, CYGB, and BRAT1, highlighted in pink).

A total of 19 genes having differences in the expression patterns between non-stress and stress chickens were common to two datasets evaluating the effects of stress in the spleen of chickens. The overall structure of the network revealed that 16 out of 19 genes were part of the network in addition to the stress condition (Fig. 1). The stress condition was directly connected in the network with only one gene, CARD19. The Markov Blanket property of the condition revealed that in addition to CARD19, CYGB was also related to the stress condition (Fig. 1, rectangle-shaped nodes).

The structure of the Bayesian networks was further explored by dividing the overall structure into smaller communities of densely connected nodes within the community but scarcely connected with nodes in other communities^43^. The application of a divisive cluster algorithm that uses the structure of the Bayesian networks as the input revealed five communities of densely connected nodes. The community of the stress condition consisted of four genes: in addition to the genes belonging to the Markov Blanket, BRAT1 and EPN3 displayed a possible interaction with the stress condition, representing a group of genes densely connected amongst themselves, but scarcely connected with the rest of the genes (Fig. 1, nodes highlighted in pink).

The Database for Annotation, Visualization, and Integrated Discovery (DAVID) was applied to explore the biological functionality of these four genes. DAVID overrepresentation analysis identified three terms, although all were not significant after adjustments for multiple tests: Calcium signaling pathway (KEGG Pathway, P-value = 0.026, Benjamini adjusted P-value = 0.28), sarcolemma (GO term, P-value = 0.045, Benjamini adjusted P-value = 1), and membrane (keyword, P-value = 0.06, Benjamini adjusted P-value = 1). The DAVID Functional Annotation Table (Table 1) shows the KEGG pathways^44^ and GO TERMS^45^ for each one of the genes being part of the community analysis of the stress condition. Terms particularly relevant to the stress condition are related to regulation of apoptotic process and caspase recruitment (CARD19), oxygen transporter activity and oxygen binding (CYGB), apoptosis process and cellular response to DNA damage stimulus (BRAT1), and endocytosis (EPN3).

**Table 1:** Functional Annotation Table provided by the Database for Annotation, Visualization, and Integrated Discovery (DAVID) corresponding to the four genes found to be in close relationship with the stressful condition.

**Caspase recruitment domain family member 19 (CARD19)**	
GO TERMS	**Regulation of apoptotic process** Integral component of membrane
**Cytoglobin (CYGB)**	
GO TERMS	Neuron projection, neuronal cell body **Oxygen transporter activity**, iron ion binding, **oxygen binding**, heme binding
**BRCA1 associated ATM activator 1 (BRAT1)**	
GO TERMS	Positive regulation of protein phosphorylation, glucose metabolic process, apoptotic process**, cellular response to DNA damage stimulus**, cell proliferation, response to ionizing radiation, cell growth, cell migration, mitochondrion localization Nucleus, cytoplasm, membrane
**Epsin 3 (EPN3)**	
KEGG PATHWAY	**Endocytosis**

In bold are highlighted terms particularly relevant to the stress condition.

## Discussion

This study was aimed at identifying a reduced number of genes closely associated with a stressful condition in the chicken as a poultry animal model. To have a more accurate approximation to the stress phenomenon, two publicly available datasets involving the measurement of gene expression in the spleen of chicken exposed to heat stress were combined into a larger dataset. After bioinformatic pre-processing and analysis, we identified a set of 19 genes common to both datasets with a differential expression pattern; these genes were used for learning the structure of the Bayesian networks. With the Bayesian network in place, its structure was divided into smaller communities of densely connected nodes. By the implementation of this approach, two genes were identified as part of the Markov Blanket property of the stress condition. In addition to these two genes, two other genes were part of the community of the condition, giving a total of 4 out of the 19 initial genes displaying a close relationship with the stress condition. Our results identified a small set of relevant genes related to stress that can be used to extract meaningful information regarding the genetics of this complex phenomenon.

Stress involves the perception of the stimulus in the immune-neuroendocrine interplay, triggering the stress response, and displaying physiological and behavioural adaptations with the aim of dealing with the stressful stimulus^2–4,6^. Heat stress has been widely studied and its effects on immune organs and immune responses have been reported^3,15,18,46^. In particular, Hirakawa et al.^47^ found that the mass of the spleen was severely affected by the exposure to heat stress^47^. Additionally, heat stress altered the structure of the spleen, having an impact on the humoral immune responses that modulate the lymphocyte populations^47^. Chickens under high environmental temperatures have also shown imbalances in the oxidant/anti-oxidant status as a consequence of the alteration of some by-products or end products of lipid peroxidation such as malondialdehyde (MDA) and thiobarbituric acid reacting substances (TBARS)^20,48^. The imbalance is created by the excess of oxidant molecules, such as reactive species containing oxygen, nitrogen, and/or chlorine, potentially affecting the structure of proteins, lipids, and DNA and RNA. Consequently, the functioning of the cell might be affected in terms of energy availability, calcium homeostasis, and mitochondrial functionality, leading to cell damage, and therefore to the survival of the cell being threatened by apoptosis or necrosis^19,49^.

The Markov Blanket property together with the community analysis revealed a total of four genes in close association with the stress condition. One of the genes, CARD19, showed a direct interaction with the stress condition, while the other three genes were part of the Markov Blanket and/or the community of the stress condition. CARD proteins belong to the family of caspase recruitment domains and they are proteins that mediate apoptosis as well as the activation of the NF- $\mathrm{\kappa \beta }$ signaling pathways^50–53^. Cytoglobin (CYGB) belongs to the globin family, whose major role is related to the provision of oxygen in different tissues and organs, in addition to a potential protective activity against reactive oxygen species^54–56^. Considering that heat stress leads to oxidative stress, cell damage, apoptosis, and immune dysfunction, CARD19 and CYGB could be identified as key genes associated with these mechanisms that chickens trigger as a consequence of the influence of the stressor. Under exposure to other stressors, such as an immune challenge with mycotoxin or hypoxic conditions, apoptotic signaling pathways were also activated in splenic cell^49,57^. Specially under hypoxic conditions, Chen et al.^49^ identified that splenic cells initiated apoptotic signaling pathways as a result of oxidative stress involving inflammatory mechanisms and the NF- $\mathrm{\kappa \beta }$ pathway^49^.

Considering the learnt structure of the Bayesian network, the further analysis of smaller communities of densely connected nodes showed that the stress condition potentially interacted with two other genes: BRAT1 and EPN3. BRCA1-associated ATM activator 1 (BRAT1) was previously identified by Qui et al.^58^ in the spleen of layer chickens undergoing an infection with avian leukosis virus (subgroup J)^58^. In humans, this gene interacts with two other genes, BRCA1 and ATM, mediating cell pathways associated with DNA damage as well as apoptosis^59–62^. Epsin-3 (EPN3) is a member of the endocytosis protein adapter gene family, and its main function is related to endocytosis^63^. Additionally, EPN3 has been identified in pathological or damaged tissues requiring wound healing^64^. In our study, it seems that both the Markov Blanket and the community of densely connected nodes of the stress condition are pointing towards key genes related to apoptosis and tissue damage. It is then plausible to highlight that when chickens are exposed to a complex phenomenon such as heat stress, one of the main immune organs, the spleen, reflects some morphological and physiological alterations as a consequence of undergoing apoptotic-related mechanisms, potentially translating into the reported suppression and dysfunction of the immune responses^32,47^.

Regarding the network approach implemented in our study, the combination of two strategies was applied with the aim of identifying genes in close relationship with the stress condition: the Markov Blanket property of Bayesian networks and the community of highly connected nodes^42,43^. Initially, the overall structure of the network, the relationships and the interaction between the given set of variables (the genes and the stressful condition) were learnt from the data. As a following step, the already learnt structure of the Bayesian network was divided into smaller groups of densely connected nodes^43^. By combining these two strategies, an initial set of 19 genes were further reduced to a small set of genes that showed a close association with the stress and that can be further studied. Among this small set of genes, the structure of the Bayesian network revealed that CARD19 showed a close interaction with the stressful condition, suggesting this gene could be explored as a potential biomarker of stress. Therefore, further research can be developed with short-term goals, such as using these genes to identify chickens raised under non-stress or stress conditions, and consequently, using them as indicators of stress, raising the alarm to monitor and manage the breeding conditions to mitigate the detrimental effects of stress on poultry production^65,66^. On the other hand, further research can have long-term goals, such as artificial selection and breeding programs in order to enhance the resilience or resistance of chicken breeds to stress, such as the Fayoumi chickens that have been used in studies as a heat stress and disease resistant breed^66–68^.

In conclusion, this study implemented a series of steps aimed at reducing an initial number of genes obtained from high-throughput technologies to a small number of genes, and unravelling their relationships and interactions. We combined two previous studies that evaluated the effects of stress on the spleen of chickens to get a more accurate approximation to the stress phenomenon. The series of steps involved the combination of: (i) bioinformatic tools to identify differentially expressed genes, (ii) Bayesian networks to learn the overall structure of the network, (iii) the Markov Blanket together with the community analysis to identify a small set of genes in close association with the stress condition, and (iv) the database for biological knowledge discovery DAVID. Such a sequence of computational approaches could be applicable to many studies of gene expression, across many measurement platforms, enabling combination of power from multiple experiments to identify of small sets of genes for further study. This work is complementary to the original studies, in that the genes we identify are not an overall picture (this would be found in those studies), but instead provide information about a small set of genes with a strong signal, across multiple studies, suggesting relation to the condition of interest. Here, the outcome of this series of steps identified two genes as being part of the Markov Blanket and two additional genes as being part of the community analysis for the stress condition in poultry. The biological processes of these four genes were related to damage and apoptosis, and they could potentially be further used as biomarkers of heat stress. The exploratory nature of our study demands future research to determine the discovered protein–protein interactions *in-vivo,* comparing the differences between chickens raised under non-stress conditions and chickens raised under stress conditions.

## Methods

### Dataset

Two datasets were collected from a publicly available data repository (Gene Expression Omnibus—GEO), under the following accession numbers: GSE119387 and GSE85434. Briefly, chickens studied in the GSE119387 dataset came from two different regions of Ethiopia: low altitude regions are hot and humid, with chickens adapted to heat conditions, and high altitude regions, on the other hand, are cooler, with chickens susceptible to heat conditions. The effects of heat stress conditions were evaluated in chickens coming from both regions, but they were raised in low altitude regions (hot and humid). Chickens studied in GSE85434 were exposed either to thermoneutral condition (25 °C—control) or to thermal treatment (35 °C—heat stress) for 3.5 h. Therefore, both datasets evaluated the effects of heat stress on gene expression in the spleen of chickens, measured by RNA-sequencing technologies. Each dataset was individually analyzed with the aim of identifying genes relevant for stress, determined by differential expression patterns between non-stress and stress conditions. The .txt files were downloaded, imported into R^69^, and pre-processed using the R package “edgeR”^70^, normalizing and removing any possible background noise associated with the data. Thereafter, the *lmfit* function, from the R package “limma”^71^, was implemented to fit a linear model according to the experimental design of each dataset. The *eBayes* function was applied to calculate the statistics that would identify the set of genes. Finally, the top highly significant genes were selected using the *topTable* function. A list of 677 and 483 relevant genes were independently identified for each dataset. To combine the power of the two studies, we examined those genes in common between the two datasets, giving a total of 19 genes shared by the two studies, and representing a manageable number of variables to learn the structure of Bayesian networks. The expression values for each gene were extracted from the datasets and were used to create the final dataset, which consisted of 19 genes and 50 individuals. This dataset was discretized into three categories, low, medium or high, applying the function *discretizeDF*, within the “arules” package^72^, following a quantile discretization distribution. The discretization of the data was implemented to reduce noise possibly related to differences between experimental designs as well as to increase the statistical power^35,40^. Once the data had been discretized, the stress condition was included as a binary variable, taking a value of 0 for chickens raised under non-stress conditions and a value of 1 for chickens exposed to the stress condition.

### Bayesian networks and community analysis

To learn the structure of Bayesian networks, the software Banjo was implemented (available for free for academic purposes from http://www.cs.duke.edu/~amink/software/banjo/)^35,37^. Banjo implements heuristic searches with two possible algorithms, Greedy or Simulated Annealing, and scores each network with a BDe score. Banjo allows the possibility of selecting the top highest scoring network or combining the n top highest-scoring networks into one consensus network as the output. In this study, the search space was explored with a total of 250 million networks, using a Simulated Annealing algorithm with local random moves as the proposer. A consensus network was built combining the top 100 high-scoring networks. Considering that Bayesian networks implement heuristic searches, adding, removing, or reversing edges with the aim of finding the highest scoring network, the final set of edges was slightly different after running the algorithm several times. Therefore, with the aim of finding the Bayesian network that best fitted the data, the following strategy was implemented to solve this challenge. The search space was explored a total of 1000 times, resulting in 1000 consensus networks. These 1000 networks were divided into 10 groups of a hundred networks (10 × 100 = 1000). Within each set of a hundred networks, all the arcs identified among these 100 networks were used to create a matrix of presence/absence. Thereafter, for each individual network, if an arc was present in the set of arcs, a value of one was assigned; while, if the arcs was absent, a value of zero was assigned. Ten absence/presence matrices were further used for calculating the weight of the arcs: the presence/absence values of each of the arcs (either one or zero) were added across networks; the weight of an arc could take values between 1 and 100. Those arcs whose weight values were equal to or higher than 50 were selected to build the network that best fitted the data. Once the consensus Bayesian networks were built, a further step was taken using the structure of the networks to implement a community analysis. The aim of this community analysis is to identify clusters of nodes densely connected amongst themselves but scarcely connected with nodes between clusters^43^. The approach implemented is similar to the one used when performing cluster analysis, with the difference that the input is a network. It uses a hierarchical divisive clustering, beginning with the structure of the network, identifying the least connected nodes between two given variables and then, removing these arcs for the next step^43^. The process is repeated several times, until the whole network is divided into smaller communities of densely connected nodes^43^. The R package “*igraph*”^73^ was implemented to identify the communities within the consensus Bayesian networks. Initially, the function *cluster_edge_betweenness* was applied to group nodes densely connected, and then the function *dendPlot* was implemented to visualize the results.

### DAVID bioinformatics database

In order to provide further insights into the biological meaning of the genes in close association with the stress condition, the database for annotation, visualization and integrated discovery (DAVID) was explored^74^. DAVID is a bioinformatic resource, publicly available (https://david.ncifcrf.gov), that combines different sources of information, such as protein–protein interactions, bio-pathways, GO terms, homology, literature, among many others, with the aim of closing the gap between a list of statistically significant genes and their functional and biological meaning^74^. DAVID implements some search algorithms to classify the genes into groups of genes that have similar annotation terms, determine which of these genes have overrepresented biological terms, and identify annotations and terms related to a particular gene^74^. DAVID provides a useful bioinformatic resource to further explore and visualize a list of genes, focusing not only on individual genes, but also on groups of genes that might be related to each other based on their annotations. In this study, we used the corresponding ENSEMBL GENE IDs as the input; the Functional Annotation Chart and the Functional Annotation Table provided by DAVID were used as the outputs. The former provides a list of overrepresented annotation terms considering the list of genes as a whole, while the latter is focused on each individual gene and provides their corresponding annotation terms.
